# Detection rate of actionable mutations in diverse cancers using a biopsy-free (blood) circulating tumor cell DNA assay

**DOI:** 10.18632/oncotarget.7110

**Published:** 2016-02-01

**Authors:** Maria Schwaederle, Hatim Husain, Paul T. Fanta, David E. Piccioni, Santosh Kesari, Richard B. Schwab, Kimberly C. Banks, Richard B. Lanman, AmirAli Talasaz, Barbara A. Parker, Razelle Kurzrock

**Affiliations:** ^1^ Center for Personalized Cancer Therapy and Division of Hematology and Oncology, UCSD Moores Cancer Center, La Jolla, CA, USA; ^2^ Guardant Health, Inc., Redwood City, CA, USA

**Keywords:** cancer, liquid biopsy, ctDNA, actionable alteration, personalized therapy

## Abstract

Analysis of cell-free DNA using next-generation sequencing (NGS) is a powerful tool for the detection/monitoring of alterations present in circulating tumor DNA (ctDNA). Plasma extracted from 171 patients with a variety of cancers was analyzed for ctDNA (54 genes and copy number variants (CNVs) in three genes (*EGFR*, *ERBB2* and *MET*)). The most represented cancers were lung (23%), breast (23%), and glioblastoma (19%). Ninety-nine patients (58%) had at least one detectable alteration. The most frequent alterations were *TP53* (29.8%), followed by *EGFR* (17.5%), *MET* (10.5%), *PIK3CA* (7%), and *NOTCH1* (5.8%). In contrast, of 222 healthy volunteers, only one had an aberration (*TP53*). Ninety patients with non-brain tumors had a discernible aberration (65% of 138 patients; in 70% of non-brain tumor patients with an alteration, the anomaly was potentially actionable). Interestingly, nine of 33 patients (27%) with glioblastoma had an alteration (6/33 (18%) potentially actionable). Overall, sixty-nine patients had potentially actionable alterations (40% of total; 69.7% of patients (69/99) with alterations); 68 patients (40% of total; 69% of patients with alterations), by a Food and Drug Administration (FDA) approved drug. In summary, 65% of diverse cancers (as well as 27% of glioblastomas) had detectable ctDNA aberration(s), with the majority theoretically actionable by an approved agent.

## INTRODUCTION

The detection and investigation of molecular alterations has increased our knowledge of oncogenic mechanisms, and led to the use of targeted cancer therapies matched to patients' specific molecular aberrations [1–7]. Specific examples include the use of EGFR tyrosine kinase inhibitors in non-small cell lung cancer (NSCLC) with an *EGFR* mutation [8], BRAF inhibitors in melanoma harboring *BRAF* mutations [9,10], or imatinib for chronic myelogenous leukemia [11,12]. Currently, most of the molecular tests are performed on archived tissues at a single time point, which is often a limiting factor. These invasive biopsies involve risks for patients, they are costly, time consuming, and may pose related complications of tissue acquisition. Samples used for testing are often from archived tissue that is several months old [1,2], which may not provide a recent picture of the molecular background of the tumor. In addition, patients with metastatic disease often have multiple involved sites, but usually have only one tumor biopsied and interrogated. While the testing might provide information about the genomic landscape of this particular site, it may not reflect the full genomic make-up of the cancer, as it has been shown that metastatic disease exhibits considerable heterogeneity [13–15]. Furthermore, it has been established that the portfolio of alterations found in tumors evolve with time. For instance, patients with non-small cell lung cancers treated with an EGFR inhibitor to target an *EGFR* mutation nearly always develop resistance, due to secondary mutations [16–18]. Therefore, molecular assays that detect these genomic changes without repeat invasive tissue biopsies are needed. One approach that could be useful is to investigate circulating tumor cell DNA (cell-free DNA) shed into the circulation or released when cancer cells die [19]. This technology has emerged rapidly, with detection of the small amounts of tumor DNA present in the blood being virtually impossible even a few years ago. More recently, circulating cell-free tumor DNA has been successfully analyzed for single gene aberrations [20–22]. Cell-free DNA assays analyzing complete exons in multiple genes via next-generation sequencing (NGS) are now becoming feasible. Here we report the results of liquid biopsies in 171 patients whose blood was analyzed for 54 genes via NGS in circulating tumor, cell-free DNA.

## RESULTS

### Analysis of control samples from healthy persons

During the technology development process, 79 healthy normal controls (source: AllCells, http://www.allcells.com) were tested and, in those, a single *TP53* R248Q mutation (heavy smoker, but no history of cancer) was seen, typical of a somatic mutation. During patient testing, samples from an additional 143 healthy persons were analyzed as controls (not blinded). These controls comprised about 60% male, with age ranging from 20-50 years old. None of these individuals had a detectable somatic mutation in the 54 gene panel (single nucleotide polymorphisms (SNPs) were commonly seen but these are ascertained as germline SNPs because they occur at close to 50% or 100% mutant allele frequencies in cell-free DNA).

### Patient characteristics

Our population comprised 171 patients with diverse cancers who had a biopsy-free next-generation sequencing ctDNA test performed on their blood. Patient's median age was 57 years old (range 19-87). There was a predominance of women over men (n=104 (61%): n=67 (39%)), and the most commonly represented cancers were lung (23%), breast (23%), and glioblastoma (19%) Table 1.

**Table 1:** Population characteristics

Parameters	Total patients, N = 171
**Gender**	
Women	104 (61%)
Men	67 (39%)
**Age (median, range)**	57.4 years (19-87)
**Turn over time^a^ (median, 95%CI)**	13 days (12-13)
**Tumor origin**	
Lung	40 (23.4%)
Breast	40 (23.4%)
Glioblastoma	33 (19.3%)
Genitourinary	10 (5.8%)
Gastrointestinal	6 (3.5%)
Unknown primary	39 (22.8%)
Other^b^	3 (1.8%)
**Number of patients with ≥ 1 alteration**	99 (58%)

a Time from blood reception to results

b Other included: melanoma, n=1; sarcoma, n=1; thymic sarcoma, n=1

### Circulating tumor DNA (ctDNA) sequencing results

**Figure 1:**
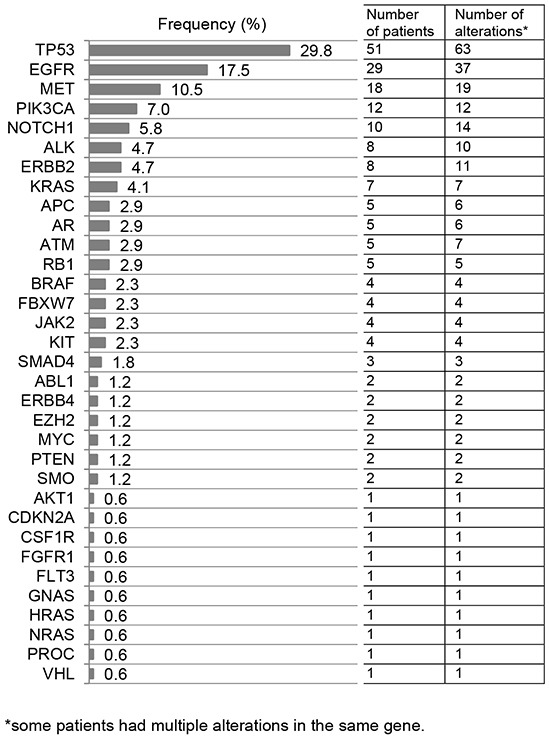
List of altered genes Overall, 211/238 (89%) alterations were mutations and 27/238 (11%) were amplifications. Of 29 patients with *EGFR* alterations, 11 (38%) had an *EGFR* amplification only; two (7%), both an *EGFR* amplification and *EGFR* mutation(s); and 16 (55%) had *EGFR* mutation(s) only. Of eight patients with *ERBB2* anomalies, two (25%) had an *ERBB2* amplification only; two (25%), both an *ERBB2* amplification and *ERBB2* mutation(s); and four (50%), only an *ERBB2* mutation. Of 18 patients with a *MET* aberration, 10 (56%) had a *MET* amplification only (10/18=56%) and eight (44%), a *MET* mutation only.

The median time from sample receipt by the testing laboratory to results was 13 days (95%CI 12-13 days; range 6-27 days). In our 171 tested patients, 238 alterations were identified, with the majority being mutations (n=211, 89%). Eleven percent of the identified alterations were amplifications (27/238), although only 3 genes were tested for copy numbers (*ERBB2, EGFR*, and *MET*). The most frequent alterations identified were *TP53* (29.8%), followed by *EGFR* (17.5%), *MET* (10.5%), *PIK3CA* (7%), and *NOTCH1* (5.8%) (Figure 1).

**Figure 2:**
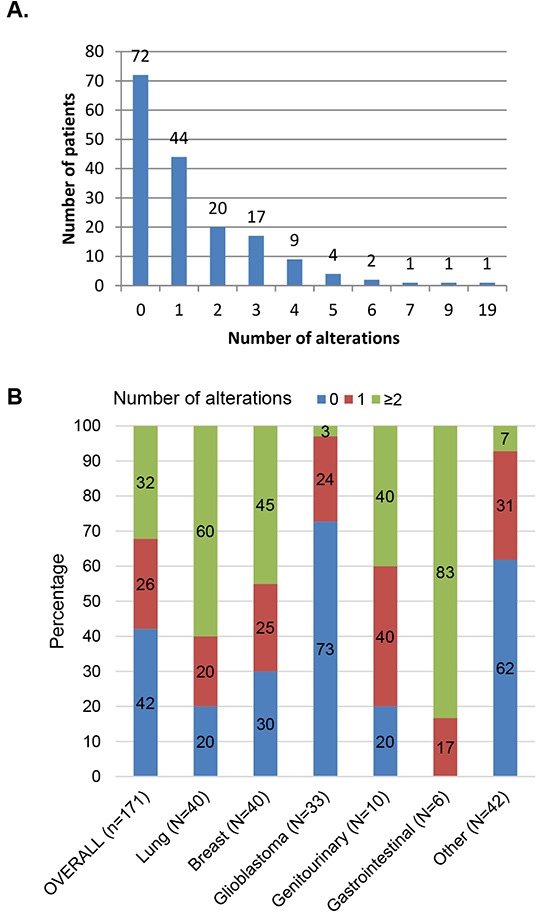
Description of the number of alterations identified in 171 patients **Panel A.** displays the number of patients per designated number of alterations (total=238 alterations; median 1 alteration per patient, range 0-19). A total of 99 patients (58%) had alterations(s). **Panel B.** describes the percentage of patients with the designated number of alterations, by histology. As an example, for patients with lung cancer: 20% had no alterations, 20% had 1 alteration, and 60% had ≥2 alterations reported. Other included: unknown primary, n=39; melanoma, n=1; sarcoma, n=1; thymic sarcoma, n=1.

Of the total, 99 patients (58%) had at least one detectable alteration(s). This includes 65% (90/138) of patients with cancers other than glioblastoma, and 27% (9/33 of glioblastoma cases). Specifically, 26% of patients had one alteration, and 32% had at least two alterations. Patients had a median of one alteration (average 1.4, range 0-19), Figure 2A. Gastrointestinal, lung, breast, and genitourinary cancers harbored the most alterations, with 83%, 60%, 45%, and 40% of cases bearing at least two alterations, respectively. Interestingly, while the majority of patients with glioblastoma (73%) did not harbor a discernible alteration, 27% (N=9/33) had an alteration (Figure 2B), most commonly *TP53* and *NOTCH1* anomalies (detected in four and three patients, respectively).

**Figure 3:**
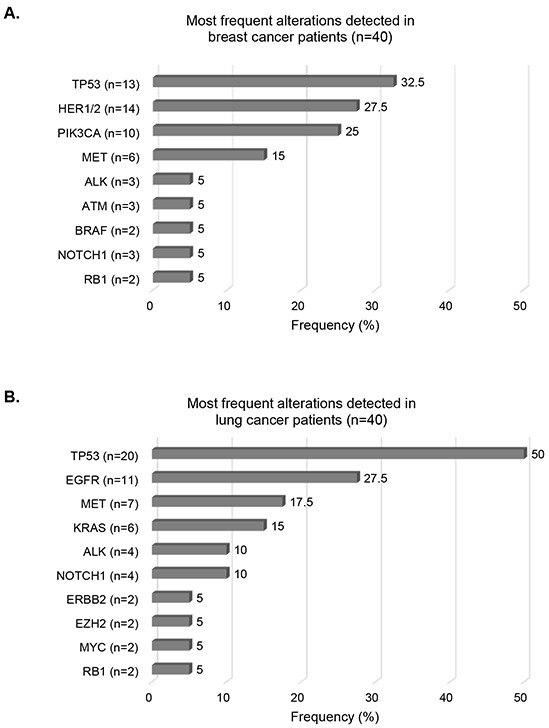
Most frequent alterations detected in breast and lung cancers Bar graphs representing frequencies of the most frequent alterations for breast cancer cases **Panel A.** and lung cancer cases **Panel B.** Alterations harbored by ≥ 2 patients have been included. Numbers into brackets indicate the number of patients with the designated alteration.

When examining the tumor types comprising the most patients (lung and breast cancers, each n=40), we found that the most frequent alterations reported were *TP53* (32.5%), *HER1/2* (27.5%), and *PIK3CA* (25%) in breast cancer cases (Figure 3A). In lung cancer cases, *TP53* alterations were detected in 50% of the cases, followed by *EGFR* (27.5%) and *MET* (17.5%) alterations (Figure 3B).

### Actionability of the detected alterations

**Figure 4:**
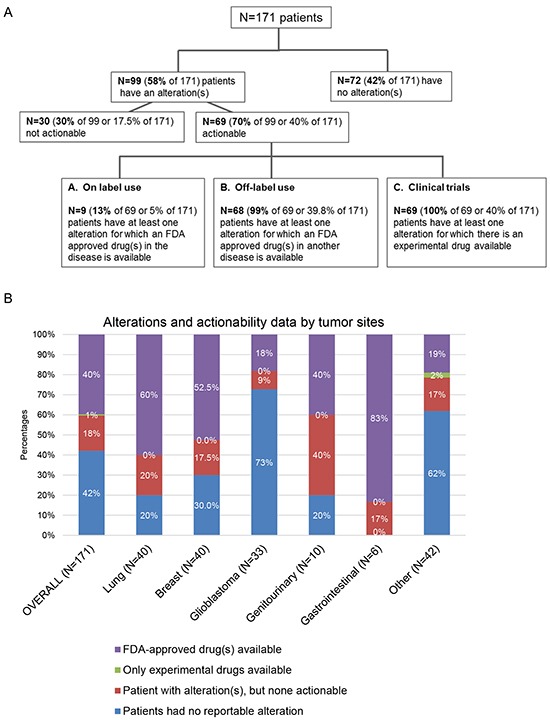
Analysis of actionability in 171 patients with diverse cancers **Panel A.** there is some overlapping as some patients might have approved agents on and off-label, as well as experimental drugs options for their disease - patients described in box A (on label use) all also have “off label use” options and are included in box B (Off-label use). Similarly, patients described in boxes A and B all also have clinical trial options and are included in box C. All patients with actionable alterations had at least one clinical trial suggested. **Panel B.** displays the percentages of actionability data by tumor sites. Other included: unknown primary, n=39; melanoma, n=1; sarcoma, n=1; thymic sarcoma, n=1.

Of the total of 171 analyzed patients, 69 had potentially actionable alterations (40% of total, 70% of the patients with alterations detected). Indeed, all these 69 patients had at least one matched experimental drug available in clinical trials. Among these 69 patients, 68 patients (99% of 69 or 40% of total patients) also had a least one matched FDA- approved drug (n=9 patients with on-label use), Figure 4 and Table 2. Of note, the majority of patients with gastrointestinal, lung, and breast cancers had actionable alterations (83%, 60%, and 52.5%, respectively)—in all the cases with an actionable aberration, the alteration could be matched to an FDA-approved drug, Figure 4B.

**Table 2:** Alterations and actionability in 171 patients with diverse cancer types

Patients cases	No reportable alteration (N, %)	Patients had alteration(s), but none actionable (N, %)	Approved drug(s) in the disease available (N, %)^b^	Approved drug(s) in another disease available (N, %)^b^	Experimental treatment^b^ (Clinical trials) available (N, %)
Histology			(On-label)	(Off-label)	
Lung (N=40)	8 (20%)	8 (20%)	5 (12.5%)	24 (60%)	24 (60%)
Breast (N=40)	12 (30%)	7 (17.5%)	2 (5%)	21 (52.5%)	21 (52.5%)
Glioblastoma (N=33)	24 (73%)	3 (9%)	0	6 (18%)	6 (18%)
Genitourinary (N=10)	2 (20%)	4 (40%)	1 (10%)	4 (40%)	4 (40%)
Gastrointestinal (N=6)	0	1 (17%)	1 (17%)	5 (83%)	5 (83%)
Other^a^ (N=42)	26 (62%)	7 (16.7%)	0	8 (19%)	9 (21%)
**OVERALL (N=171)**	**72 (42%)**	**30 (17.5%)**	**9 (5%)**	**68 (39.8%)**	**69 (40%)**

a Other included: unknown primary, n=39; melanoma, n=1; sarcoma, n=1; thymic sarcoma, n=1.

b Note: there is some overlapping as some patients had approved agents on and off-label, as well as experimental drugs options for their disease. In total, 68 patients (39.8%) had one or more approved drug(s) as an option: 9 patients had both on- and off-label options, 59 had off-label only.

On the other hand, 102 patients (60%) had no actionable alterations. In 72 of the 102 patients (70.6%) (or 42% of the total of 171 patients), there was no alteration detected. In addition, some patients (n=30, 17.5% of the 171 patients) had alterations, but they were not theoretically actionable with currently available drugs, either approved or investigational (Figure 4).

## DISCUSSION

Herein, we studied the molecular alterations identified in 171 patients with diverse cancers using targeted next-generation sequencing that analyzed circulating tumor DNA (ctDNA) extracted from plasma. We found that 58% of our patients demonstrated at least one molecular alteration, and 32% had two or more alterations. The most frequent alterations were *TP53* mutations, detected in nearly 30% of our patients, similar to previous reports wherein tissue was interrogated [23]. Of interest, the frequency of some alterations, such as *MET* in breast and lung tumors, was higher than previously reported, though the rates reported vary widely by study [24–27]. The higher rates of *MET* anomalies in our study could be due to the advanced state of the patients who usually undergo ctDNA testing, the relatively small number of patients with each disease type in our study, or the propensity for DNA bearing *MET*-related alterations to be shed into the blood.

Forty percent of patients had a potentially actionable alteration. Among the 99 patients with alterations detected, 70% had a potentially actionable alteration. Of note, three recent actionability studies performed on tumor samples [28–30] (not ctDNA) report that 83-90% percent of patients had at least one actionable alteration, which is higher than in earlier reports, perhaps because more comprehensive panels are now in use for tissues, and more targeted drugs have been approved and entered clinical trials. The ctDNA assay's actionability was based on a panel of 54 genes (point mutations tested for all genes and copy numbers assessed in three of the 54 genes) (other genomic alterations such as indels and fusions were not included at that time). In contrast, the previous publications on tissue-based studies utilized larger panels of about 200 genes assessed by NGS [28–30]. Furthermore, the patient populations examined, as well as the sensitivity of ctDNA versus tissue assays may be other factors that account for these differences. Indeed, in our study, 42% of patients did not have any detectable alteration. This was most prominent for glioblastoma, where 73% of patients (N = 33 total tested) had no discernible alteration in their ctDNA. Of interest in this regard, Bettegowda et al. [31] demonstrated the ability to detect any ctDNA (albeit without sequencing the genes in the ctDNA) in over 75% of 640 patients with various cancer types, but in less than 50% of primary brain, renal, prostate, or thyroid cancers, suggesting that physical obstacles such as the blood-brain barrier and mucin could prevent ctDNA from entering the circulation [32]. For patients with primary brain cancers, cerebral spinal fluid may serve as an alternative “liquid biopsy” by enabling a more direct measurement of circulating DNA [33]. On the other hand, it is interesting that 27% of our patients with glioblastoma did have a discernible aberration on ctDNA testing, suggesting the possibility that, as this technology improves and incorporates highly prevalent alterations such as *EGFR* vIII indels, it may become usable for even higher percentages of these patients.

Overall, 65% of patients with malignancies other than brain tumors had at least one identifiable anomaly in their ctDNA. Sixty-eight patients (40%) had abnormalities that could be prosecuted by at least one drug that was approved for another disease (off-label use); and 9 patients (5%) had at least one approved agent in their disease (on-label use). Overall, 70% of patients with alteration(s) had an aberration potentially actionable by an experimental agent in clinical trials or by an approved agent. Previous experience suggests that patient eligibility for these clinical trials or their conduct at a limited number of enrolling sites, as well as difficulty obtaining coverage for off-label drug use, might limit patients' access to cognate medications [2].

Actionability by drugs that were FDA-approved was common in breast (52.5%) and lung cancers (60%). For lung cancers, *EGFR* alterations were frequent alterations; they are actionable with approved drugs (since several EGFR inhibitors are approved, including erlotinib, which is authorized for lung cancer). For breast cancers, most of the alterations actionable with an approved drug were in the *PTEN/PI3K/mTOR* axis (with several mTor inhibitors approved, including everolimus, which is on-label for breast cancer) or in the *HER* pathway.

There were several limitations to this study. First, it included a limited number of patients in each histology. Second, clinical annotation was not available since the database was de-identified. Third, the definition of “actionable” and the level of evidence needed for such a determination is a matter of debate and in constant evolution [34]. Fourth, the use of tissue-based next generation sequencing as a comparison to establish clinical utility was not accessible for this group of de-identified patients. However, because many of our patients were on treatment, it is important to note that concordance to tissue-based NGS is challenged by the fact that ctDNA is a dynamic measure and the original oncogenic driver mutations may become undetectable in the plasma of patients that are responding, or conversely, new resistance mutations may arise in ctDNA that were not seen in the original tissue biopsy [35–37]. Further, ctDNA tests may measure shed DNA from multiple metastases. On the other hand, ctDNA tests may not be sensitive enough to detect some tissue NGS alterations. Additional studies will need to perform in-depth analyses of the concordance between tissue and ctDNA molecular results to better understand the observations from each test. Finally, whether or not the patients would have responded to these drugs could not be addressed in this study, and will require further investigation.

In conclusion, “liquid biopsies” are non-invasive, and have several advantages compared to tissue biopsies. Most importantly, they require only a small amount of blood, rather than a biopsy that can be invasive, painful, and, in some cases, have complications. Evaluation of ctDNA obtained from liquid biopsies is therefore also amenable to repeat sampling. Furthermore, obtaining a blood sample is efficient and inexpensive compared to obtaining a tissue biopsy. Indeed, biopsies often cost thousands of dollars; transthoracic and transbronchial lung needle biopsies have been reported to cost over $14,000 on average, based on a population-based national Medicare sample [38]. Finally, it is now known that there is molecular heterogeneity within and between tumors in the same patient [14]. Therefore, theoretically, ctDNA results may reflect genomic aberrations in DNA shed from multiple metastatic sites. Our observations suggest that, in our population, 40% of patients (69/171) carried potentially actionable aberrations; almost all of them had an aberration that could be targeted by an approved drug (n = 68/69). However, only a minority of individuals (9 patients) had aberrations that could be targeted by drugs approved for their type of cancer (on-label). This biopsy-free test has intrinsic clinical utility by obviating the need for repeat invasive-tissue biopsies at the time of progression of a visceral malignancy. In cases where the ctDNA-based NGS panel detects no genomic alterations, an invasive tissue biopsy could then be considered to evaluate the genomic status of the tumor. The rate of actionability for the NGS test described here may further increase with more comprehensive panels. To extend evidence of clinical utility, the value of ctDNA-based, multi-panel gene assays, such as those used in this study, in monitoring patients and predicting tumor response merits investigation in prospective trials.

## MATERIALS AND METHODS

### Patients

We retrospectively reviewed the liquid biopsy results of 171 consecutive de-identified patients with diverse cancers who were seen at UC San Diego Moores Cancer Center. Blood samples were collected between June, 2014 and January 2015. Analysis was performed per UCSD IRB exempt approval for study of pre-existing de-identified data. Analysis of usage patterns of liquid biopsies at our institution indicates that about 95% of patients who have had these tests performed have advanced or metastatic disease. In addition, two series of healthy volunteers were tested as controls (N = 79 followed by N = 143) (source: AllCells, http://www.allcells.com).

### Next generation sequencing

Next generation digital sequencing was performed by Guardant Health (Guardant360, www.guardanthealth.com/guardant360/), a Clinical Laboratory Improvement Amendments (CLIA)-certified and College of American Pathologists (CAP)-accredited clinical laboratory (Guardant Health, Inc.). At the time of this study, this test identifies potential tumor-related genomic alterations via complete exon sequencing of 54 cancer-related genes including amplifications in *ERBB2*, *EGFR*, and *MET* through analysis of cell-free DNA extracted from plasma (extracted from two blood tubes), Supplementary Table S1. This circulating tumor DNA assay has high analytic sensitivity (detects single molecules of somatic tumor DNA in 10 mL blood samples), high clinical sensitivity (detects 85%+ of the single nucleotide variants detected in tissue in advanced cancer patients) (for stage III and IV solid cancers) and analytic specificity (>99.9999%) (Sensitivity was determined by comparing 165 sequential matched plasma and tissue samples (Guardant Health, data on file). Specificity >99.9999% represents the analytic specificity, calculated from a controlled study of 20 samples comparing to exome sequencing of basepair calls for all 78,000 base pairs in the panel (genomic DNA analyzed by an independent CLIA-licensed and CAP-accredited clinical laboratory). A high degree of specificity is critical to eliminate the false positives (noise) that otherwise accompany sequencing DNA at very low concentrations over long targeted regions (in this case ∼78,000 base pairs per sample). All cell-free DNA is sequenced, including the germline cell-free DNA that is derived from leukocyte lysis and the somatic ctDNA. Single nucleotide variants are quantitated as mutant allele fraction (MAF) which is the number of ctDNA fragments divided by the number of wild type DNA fragments that overlap the same mutated nucleotide base position. Gene amplifications are reported as absolute gene copy number in plasma. In each sequencing run, a normal control sample is included (Guardant360 digital sequencing panel, Guardant Health Inc, data on file).

### Definition of actionability

Actionability implies that the protein product of a genomic abnormality can be impacted by a specific targeted drug. An actionable alteration was defined as an alteration that was either the direct target (such as an EGFR inhibitor targeting an *EGFR* mutation), or a pathway component (such as an mTOR inhibitor for a *PIK3CA* mutation (since mTor is downstream of PIK3CA)) that could be targeted by at least one approved (by the Food and Drug Administration) or investigational drug in a clinical trial. (Actionability determination was based on the ability of drugs that are small molecule inhibitors to impact the aberration, with low IC50 against the product of the aberration or effectors not more than four signals removed from the gene aberration or its product; if an altered gene product could be targeted by an antibody whose primary target was the altered gene product, it was also considered actionable). Actionability was crosschecked by two investigators, including the senior investigator (RK) [34].

### Data extraction and analysis

Demographic information such as gender, age, primary tumor site, as well as the dates of sample reception, dates of results, list of alterations and actionability data (included: number of actionable alterations, and more specifically, the number of alterations with an approved drug available in the disease (on-label use), the number of alterations with an approved drug in another disease (off-label use), and the number of alterations with experimental drug(s) available (clinical trials)) were extracted from the reports and analyzed.

Most of the statistical analysis was descriptive in nature. When appropriate, median and 95% confidence intervals (95% CI) or range were reported. The sample size was determined by the available patients with genetic testing information. Analysis performed by author MS using SPSS version 22.0.

## SUPPLEMENTARY TABLE


