# Supplementary material for: Detection rate of actionable mutations in diverse cancers using a biopsy-free (blood) circulating tumor cell DNA assay
Source: Oncotarget. 2016 Feb 1;7(9):9707–17. doi: 10.18632/oncotarget.7110 (PMC4891078; doi:10.18632/oncotarget.7110)
Supplement: Supplementary file 1 [file oncotarget-07-09707-s001.pdf]

SUPPLEMENTARY TABLE

Supplementary Table S1: Guardant360 54 gene cell-free DNA NGS panel

ABL1	*AKT1*	***ALK***	***APC***	***AR***	*ATM*
BRAF	*CDH1*	***CDKN2A***	*CSF1R*	*CTBBB1*	***EGFR****
ERBB2*	*ERBB4*	*EZH2*	***FBXW7***	*FGFR1*	*FGFR2*
FGFR3	*FLT3*	*GNA11*	*GNAQ*	*GNAS*	*HNF1A*
HRAS	*IDH1*	*IDH2*	*JAK2*	*JAK3*	*KDR*
KIT	***KRAS***	***MET****	*MLH1*	*MPL*	***MYC***
NOTCH1	*NPM1*	***NRAS***	*PDGFRA*	***PIK3CA***	*PTPN11*
PTEN	***PROC***	***RB1***	*RET*	*SMAD4*	*SMARCB1*
SMO	*SRC*	*STK11*	*TERT*	***TP53***	*VHL*

Complete exon coverage for genes in bold. Unbolded genes had critical exon coverage.
*Copy number amplification of *EGFR*, *ERBB2* (HER2) and *MET* genes was done (in addition of mutations (complete exon coverage)).
